# A clinical scoring system for predicting tumor recurrence after percutaneous radiofrequency ablation for 3 cm or less hepatocellular carcinoma

**DOI:** 10.1038/s41598-021-87782-y

**Published:** 2021-04-15

**Authors:** Yong Zhu He, Kun He, Rui Qin Huang, Li Wen Liu, Shao Wei Ye, Jun Lin Qian, Peng Peng, Qi Jie Luo, Ze Liang Wang, Ze Min Hu

**Affiliations:** 1 Department of Hepatobiliary Surgery, Zhongshan Hospital Affiliated to Sun Yat-Sen University, Zhongshan City, 528400 Guangdong Province China; 2 Guangdong Medical College, Zhanjiang City, 524002 Guangdong Province China

**Keywords:** Cancer, Biomarkers, Oncology, Risk factors

## Abstract

Preoperative prediction of tumor recurrence after radiofrequency ablation (RFA) in patients with early hepatocellular carcinoma (HCC) is helpful for clinical decision-making before treatment. A total of 162 patients with HCC of 3 cm or less who were completely ablated by percutaneous RFA were divided into a derivation cohort (n = 108) and a validation cohort (n = 54). Based on X-Tiles software, Kaplan–Meier curve analysis and COX multivariate analysis to obtain valuable predictive indicators, a clinical scoring system for predicting tumor recurrence was established. In the verall cohort, derivation cohort and validation cohort, we found circulating tumor cells (CTC) > 2/3.2 mL, alpha-fetoprotein (AFP) > 20 ng/mL, and des-γ-carboxyprothrombin (DCP) > 40 mAU/mL, maximum tumor diameter > 20 mm, and the number of multiple tumors (≥ 2) are independent risk factors affecting tumor recurrence. Each independent risk factor was assigned a score of 1 to construct a predictive clinical scoring system, and X-Tiles software was used to divide the clinical score into a low-risk group (0 score–1 score), a medium-risk group (2 scores–3 scores), and a high-risk group (4 scores–5 scores). The cumulative tumor recurrence rates of patients in the low-risk group, middle-risk group, and high-risk group in 1 year, 2 years, and 3 years were 19.4%/27.5%/30.9%, 37.0%/63.2%/79.9% and 68.2%/100%/100%, respectively (Low-risk group vs medium-risk group: *P* < 0.001; medium-risk group vs high-risk group:* P* < 0.001). This clinical scoring system can predict the prognosis of patients with HCC of 3 cm or smaller undergoing percutaneous RFA, which has certain application value for making preoperative clinical decisions.

## Introduction

As the malignant tumor with the highest incidence of liver, hepatocellular carcinoma (HCC) is also one of the five most common tumors in the world, ranking third in the cause of death from malignant tumors^1,2^. According to the joint research guidelines of the European Association for the Study of the Liver (EASL) and the American Association for the Study of Liver Diseases, the recommended treatments for early liver cancer include hepatectomy and radiofrequency ablation (RFA). Especially in some countries with lack of liver donors and limited resources, hepatectomy or RFA has become the main treatment for early liver cancer^3,4^. RFA is widely regarded as a safe and effective treatment method. Compared with hepatectomy, it has the advantages of being economical, convenient, less invasive, less complications and shorter hospital stay^5,6^.

RFA is performed after considering tumor size and the number of tumors. However, the status of serological HCC markers in regard to RFA has not been considered until now^7–9^. Previous estimations of two HCC markers, Alpha-fetoprotein (AFP) and Des-γ carboxyl prothrombin (DCP), were reported to be useful for predicting prognosis and pathological invasiveness in surgical cases^10,11^. Therefore, the prognostic outcomes of the patients who underwent RFA also might be influenced by the positive conditions of these serological HCC markers^5,12,13^. In addition, the presence of circulating tumor cells (CTC) that have detached from the primary lesion and entered the blood circulation before treatment is also considered to be one of the main sources of recurrence and metastasis after liver cancer surgery, which has become an important entry point to study the process and mechanism of tumor metastasis and recurrence^14^. In this context, our team analyzed the risk factors for tumor recurrence after RFA in patients with HCC of 3 cm or less, and designed a preoperative clinical scoring system based on all independent risk factors to guide clinical strategies.

## Materials and methods

### Patients and study design

A retrospective analysis of 162 patients with HCC who received percutaneous RFA at Zhongshan Hospital Affiliated to Sun Yat-sen University from September 2015 to September 2019. HCC mainly depends on two typical imaging findings of dynamic contrast-enhanced computed tomography (CT) combined with contrast-enhanced magnetic resonance imaging (MRI) as the diagnostic criteria. For the imaging findings of atypical HCC, puncture biopsy was performed to confirm the diagnosis. At the same time, the level of tumor markers and the history of hepatitis virus infection are also supplementary factors for the diagnosis of HCC. The reasons for choosing RFA include the difficult location of the tumor requiring extensive hepatectomy, the size of the tumor, the number of tumors, liver function, and refusal to undergo hepatectomy. Participants included criteria: (1) preoperative liver imaging examination was in accordance with the maximum lesion diameter ≤ 3 cm and the number of lesions ≤ 3; (2) No macrovascular invasion, lymphatic metastasis or extrahepatic metastasis were found in imaging examination; (3) there was no preoperative hepatectomy, transcatheter hepatic arterial chemoembolization, radiotherapy and chemotherapy; (4) there was no previous history of other malignant tumors or severe other organ lesions. (5) All patients were ablated completely by CT after operation, and the definition of complete ablation was that there was at least ablation edge of 5 mm around the whole tumor, and CT showed no enhancement area in arterial phase and no defect area in portal phase. (6) All patients had complete data and complete follow-up. We established a derivative cohort and a verification cohort from random allocation of all patients, of which 108 patients were included in the derivation cohort cohort (n = 108), and 54 patients were included in the validation cohort (n = 54). The Institutional Review Board of Zhongshan Hospital Affiliated to Sun Yat-sen University approved this single-center retrospective study. All participants gave their informed consent to collect their data.

### Preoperative clinical characteristics

All relevant examinations of patients are completed within 1 week before treatment. Among them, the serum AFP level was measured by the chemical luminescence immunoassay (Clia, ADVIA Centaur AFP assay, Siemens Healthcare Diagnostics, Deerfield, IL, USA), and the DCP level was measured by the enzyme-linked immunosorbent assay (ELISA, Haicatch PIVKA-II, Sanko Junyaku Co.,Tokyo, Japan). In addition, the detection of circulating tumor cells was identified by Cyttel method (Jiangsu, China), which includes negative immunomagnetic particle method and immunofluorescence in situ hybridization (im-FISH). The former mainly uses immunomagnetic particles as the carrier, through the principle of antigen–antibody reaction, combined with centrifugation technology, to remove white blood cells from the blood in vitro so as to separate rare cells from the blood. In the latter, the samples were fixed on glass slides, dehydrated with ethanol, dried, and then hybridized with chromosome centromere probe No. 1 and chromosome centromere probe No. 8. Finally, 4-diamidine-2-phenylindole (DAPI) staining was added to seal the samples, and the circulating tumor cells were observed and counted under fluorescence microscope^15,16^.

### RFA methods

All operations were performed by a surgeon with more than 10 years of experience with the assistance of a senior radiologist. RITA 1500X radiofrequency generator (AngioDynamics, Inc. Latham, New York, USA) was used and the corresponding RFA needle was selected according to different conditions for percutaneous RFA. Under the guidance of CT, the location of the tumor was determined, and the radiofrequency needle was inserted into the focus of the patient after local anesthesia, and then the temperature was set at 95 ℃ and 100 ℃ to treat 15 min. According to the actual situation at that time, the tumor was ablated many times with multiple overlapping points, and it was confirmed that the ablation scope covered the whole tumor. Finally, it was confirmed by CT that the tumor was ablated completely after RFA. When any area adjacent to or within the ablation site shows arterial phase enhancement area and portal phase defect area on dynamic CT, re-RFA is performed immediately until the tumor is completely ablated.

### Follow-up

All patients were followed up in the outpatient clinic. Patients were reviewed once a month for CT or MRI of the abdomen within the first 3 months after RFA treatment, then every 3 months from 3 months to 1 year, and then every 6 months thereafter. During the follow-up period, the patient's tumor recurrence was recorded. The standard of relapse was that the imaging examination found and confirmed tumor progression in the ablation site, new nodules outside the ablation site, or extrahepatic metastatic lesions. Patients were followed up until the recurrence criteria were reached or the study deadline was the end point of follow-up, and the study deadline was April 1, 2020.

### Statistical analysis

For 3 cm or smaller HCC, we propose that the cutoff values of maximum tumor diameter, AFP and DCP are 20 mm, 20 ng/mL and 40 ng/mL based on previous studies^5,13,17^. There are few reports on the prognostic significance of preoperative CTC in HCC after percutaneous RFA, so the X-Tiles3.6.1 software (http://tissuearray.org/) was used to evaluate the best cutoff value of CTC. However, there are few reports on the prognostic significance of preoperative CTC in HCC after percutaneous RFA, so the X-Tiles 3.6.1 software (http://tissuearray.org/) was used to evaluate the best cutoff value of CTC. Other consecutive data are classified according to exceeding the normal value, while age is classified according to the median (≈ 60 years). SPSS25.0 statistical software (IBM Corp, Armonk, NY, USA) was used to analyze all the following data: Chi-square test was used to compare classification variables in the relationship between CTC and clinical factors. The cumulative tumor recurrence rate among different groups was evaluated by Kaplan–Meier method, and the difference of tumor cumulative recurrence rate was compared by log-rank test. In univariate analysis, the variables with *P* < 0.1 were used for multivariate Cox proportional hazard analysis. All P values were obtained by double-tail test, *P* < 0.05 was considered to be statistically significant.

### Ethics declarations

The study was approved by the Ethics Committee of Zhongshan Hospital Affiliated to Sun Yat-sen University and conforms to the principles of the Declaration of Helsinki. And it has been confirmed to obtain written informed consent from all subjects.

## Results

### Patient characteristics

**Figure 1:**
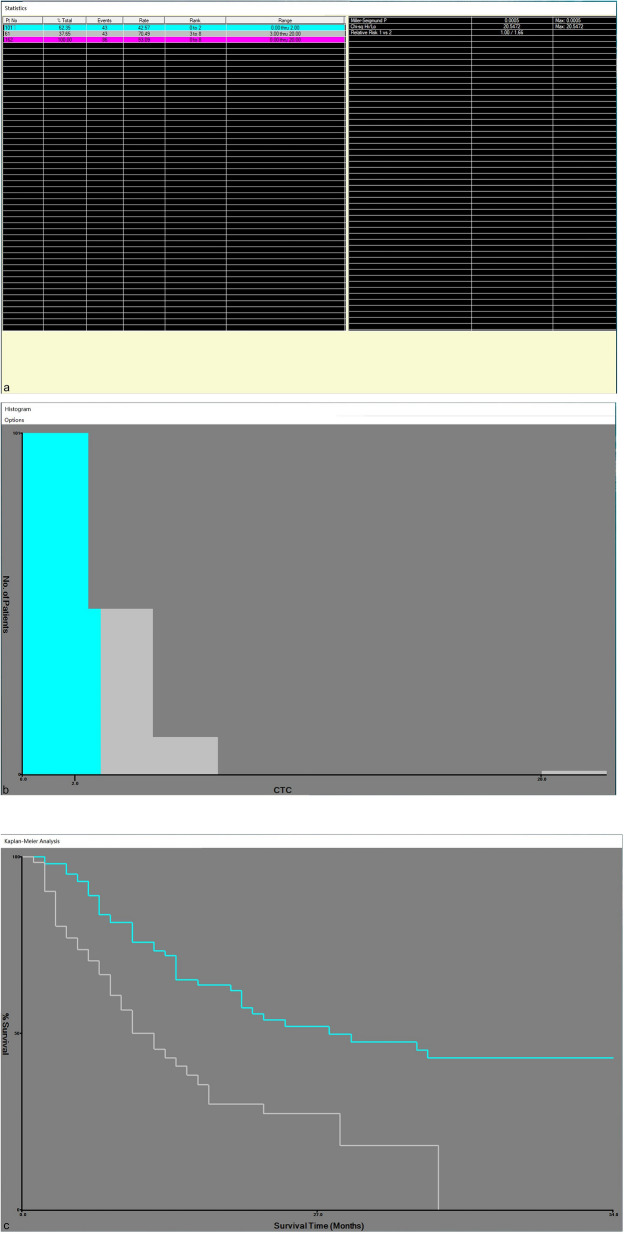
X-tile plots of preoperative CTCs and tumor-free survival of patients with HCC after percutaneous RFA. X-tile plots showing χ^2^ values with cut-off points to generate low- and high-CTC subgroups. (**a**) The optimal cut-off value of the CTCs was 2/3.2 mL at the maximum χ^2^ value of 20.5472. (**b)** Histogram of the entire cohort divided into low-CTC and high-CTC subgroups according to the optimal cut-off value of 2/3.2 mL. Blue bars represent the low-CTC group, and grey bars represent the high CTC group. (**c**) Kaplan–Meier plot of tumor-free survival in groups stratified using the optimal cut-off value of CTCs. Blue curves represent the low-CTC group, and grey curves represent the high-CTC group. RFA Radiofrequency ablation; HCC Hepatocellular carcinoma; CTC Circulating tumor cells.

As shown in Table 1, 162 patients with HCC treated by percutaneous RFA included 136 males and 26 females, with a mean age of 58.15 ± 10.61 years (median age 59 years, range 33–79 years). Among them, the derivation cohort contains 91 males and 17 females with an average age of 58.44 ± 10.27 years; while the validation cohort contains 45 males and 9 females with an average age of 57.57 ± 11.33 years. Overall, there were no significant differences in clinical characteristics between the derivation cohort and the validation cohort (*P* > 0.05). In addition, X-Tiles software was used to identify 2/3.2 mL as the best cutoff value and divided into low CTC group and high CTC group (Fig. 1a–c). Among the 162 patients, the incidence of high AFP levels, maximum tumor diameter > 20 mm and tumor numbers ≥ 2 was higher in the high CTC group in Table 2 (*P* = 0.007, *P* = 0.018, *P* < 0.001).

**Table 1:** Clinical characteristics of derivation cohort and validation.

Clinical characteristics	Overall cohort (n = 162)	Derivation cohort (n = 108)	Validation cohort (n = 54)	*P* value
Age (years)				0.502
≤ 60	90 (55.6%)	58 (53.7%)	32 (59.3%)	
> 60	72 (44.4%)	50 (46.3%)	22 (40.7%)	
Sex				0.880
Male	136 (80.4%)	91 (84.3%)	45 (83.3%)	
Female	26 (16.0%)	17 (15.7%)	9 (16.7%)	
Hepatitis B virus infection				0.730
Yes	143 (88.3%)	96 (88.9%)	47 (87.0%)	
No	19 (11.7%)	12 (11.1%)	7 (13.0%)	
Liver cirrhosis				1.000
Yes	114 (70.4%)	76 (70.4%)	38 (83.3%)	
No	48 (29.6%)	32 (29.6%)	16 (29.6%)	
Child–Pugh				0.371
A	135 (83.3%)	88 (81.5%)	47 (87.0%)	
B	27 (16.7%)	20 (18.5%)	7 (13.0%)	
CTC (n/3.2 mL)				0.361
≤ 2/3.2 mL	100 (61.7%)	64 (59.3%)	36 (66.7%)	
> 2/3.2 mL	62 (38.3%)	44 (40.7%)	18 (33.3%)	
AFP (ng/mL)				1.000
≤ 20	114 (70.4%)	76 (70.4%)	38 (70.4%)	
> 20	48 (29.6%)	32 (29.6%)	16 (29.6%)	
DCP (mAU/mL)				0.912
≤ 40	79 (48.8%)	53 (49.1%)	26 (48.1%)	
> 40	83 (51.2%)	55 (50.9%)	28 (51.9%)	
TB (μmol/L)				0.151
≤ 20.4	111 (68.5%)	70 (64.8%)	41 (75.9%)	
> 20.4	51 (31.5%)	38 (35.2%)	13 (24.1%)	
ALT (U/L)				0.449
≤ 50	136 (84.0%)	70 (64.8%)	41 (75.9%)	
> 50	26 (16.0%)	38 (35.2%)	13 (24.1%)	
AST (U/L)				0.318
≤ 40	118 (72.8%)	76 (70.4%)	42 (77.8%)	
> 40	44 (27.2%)	32 (29.6%)	12 (22.2%)	
Alb (g/L)				0.340
≤ 35	34 (21.0%)	25 (23.1%)	9 (16.7%)	
> 35	128 (79.0%)	83 (76.9%)	45 (83.3%)	
GGT (U/L)				0.819
≤ 60	101 (62.3%)	68 (63.0%)	33 (61.1%)	
> 60	61 (37.7%)	40 (37.0%)	21 (38.9%)	
ALP (U/L)				0.545
≤ 125	136 (84.0%)	92 (85.2%)	44 (81.5%)	
> 125	26 (16.0%)	16 (14.8%)	10 (18.5%)	
CR (μmol/L)				0.666
≤ 104	157 (96.9%)	104 (96.3%)	53 (98.1%)	
> 104	5 (3.1%)	4 (3.7%)	1 (1.9%)	
INR				0.217
≤ 1.20	150 (96.9%)	102 (94.4%)	48 (88.9%)	
> 1.20	12 (3.1%)	6 (5.6%)	6 (11.1%)	
Maximum tumor diameter (mm)				0.316
≤ 20	87 (53.7%)	55 (50.9%)	32 (59.3%)	
> 20	75 (46.3%)	53 (49.1%)	22 (40.7%)	
Number of tumors				0.180
Single	90 (55.6%)	64 (59.3%)	26 (48.1%)	
Multiple (≥ 2)	72 (44.4%)	44 (40.7%)	28 (51.9%)	

CTC, Circulating tumor cell; AFP, Alpha-fetoprotein; DCP, Des-γ-carboxy prothrombin; TB, Total bilirubin; ALT, Alanine aminotransferase; AST, Aspartate aminotransferase; Alb, Albumin; GGT, Gamma-glutamyltransferase; ALP, Alkaline phosphatase; CR, Creatinine; INR, International normalized ratio.

**Table 2:** Relationship between CTCs and clinical characteristics.

Clinical characteristics, n (%)	CTC ≤ 2/3.2 mL	CTC > 2/3.2 mL	*P* value
	n = 100 (61.7%)	n = 62 (38.3%)	
Age (years)			0.427
≤ 60	58 (58.0%)	32 (51.6%)	
> 60	42 (42.0%)	30 (48.4%)	
Sex			0.644
Male	85 (85.0%)	51 (82.3%)	
Female	15 (15.0%)	11 (17.7%)	
Hepatitis B virus infection			0.100
Yes	85 (85.0%)	58 (93.5%)	
No	15 (15.0%)	4 (6.5%)	
Liver cirrhosis			0.896
Yes	70 (70.0%)	44 (71.0%)	
No	30 (30.0%)	18 (29.0%)	
Child–Pugh			0.312
A	81 (81.0%)	54 (87.1%)	
B	19 (19.0%)	8 (12.9%)	
AFP (ng/mL)			**0.007**
≤ 20	78 (78.0%)	36 (58.1%)	
> 20	22 (22.0%)	26 (41.9%)	
DCP (mAU/mL)			0.690
≤ 40	50 (50.0%)	29 (46.8%)	
> 40	50 (50.0%)	33 (53.2%)	
TB (μmol/L)			0.597
≤ 20.4	67 (67.0%)	44 (71.0%)	
> 20.4	33 (33.0%)	18 (29.0%)	
ALT (U/L)			0.367
≤ 50	86 (86.0%)	50 (80.6%)	
> 50	14 (14.0%)	12 (19.4%)	
AST (U/L)			0.718
≤ 40	72 (72.0%)	43 (69.4%)	
> 40	28 (28.0%)	19 (30.6%)	
Alb (g/L)			0.996
≤ 35	21 (21.0%)	13 (21.0%)	
> 35	79 (79.0%)	49 (79.0%)	
GGT (U/L)			0.434
≤ 60	60 (60.0%)	41 (66.1%)	
> 60	40 (40.0%)	21 (33.9%)	
ALP (U/L)			0.194
≤ 125	81 (81.0%)	55 (88.7%)	
> 125	19 (19.0%)	7 (11.3%)	
CR (μmol/L)			1.000
≤ 104	97 (97.0%)	60 (96.8%)	
> 104	3 (3.0%)	2 (3.2%)	
INR			0.215
≤ 1.20	95 (95.0%)	55 (88.7%)	
> 1.20	5 (5.0%)	7 (11.3%)	
Maximum tumor diameter (mm)			**0.018**
≤ 20	61 (61.0%)	26 (41.9%)	
> 20	39 (39.0%)	36 (58.1%)	
Number of tumors			**< 0.001**
Single	54 (62.8%)	23 (39.0%)	
Multiple	32 (37.2%)	36 (61.0%)	

CTC, Circulating tumor cell; AFP, Alpha-fetoprotein; DCP, Des-γ-carboxy prothrombin; TB, Total bilirubin; ALT, Alanine aminotransferase; AST, Aspartate aminotransferase; Alb, Albumin; GGT, Gamma-glutamyltransferase; ALP, Alkaline phosphatase; CR, Creatinine; INR, International normalized ratio.

### Univariate and multivariate analysis of tumor recurrence

The summary of univariate and multivariate analysis is shown in Table 3. COX multivariate analysis showed that CTC (≤ 2/3.2 mL vs > 2/3.2 mL) (HR:1.820; 95%CI:1.168–2.834; *P* = 0.008), AFP (≤ 20 ng/mL vs > 20 ng/mL) (HR:1.750; 95%CI:1.105–2.771; *P* = 0.017), DCP (≤ 40 mAU/mL vs > 40 mAU/mL) (HR:1.936; 95%CI:1.219–3.075; *P* = 0.005), maximum tumor diameter (≤ 20 mm vs > 20 mm) (HR:1.813; 95%CI:1.119–2.939; *P* = 0.016) and tumor number (single vs multiple) (HR:1.766; 95%CI:1.082–2.883; *P* = 0.023) were independent risk factors for tumor recurrence after RFA of HCC.

**Table 3:** Univariate and multivariate analysis of Cox regression model for recurrence of HCC after RFA.

Clinical characteristics	Univariate		Multivariate	
	HR (95%CI)	*P*	HR (95%CI)	*P*
Age (years), ≤ 60 vs > 60	1.042 (0.680–1.598)	0.850		
Sex, male vs female	0.823 (0.469–1.442)	0.496		
Hepatitis B virus infection, Yes or No	1.130 (0.546–2.340)	0.741		
Liver cirrhosis, Yes or No	1.044 (0.647–1.684)	0.859		
Child–Pugh A vs B	0.517 (0.274–0.977)	0.042	0.605 (0.318–1.153)	0.127
CTC, ≤ 2/3.2 mL vs > 2/3.2 mL	2.497 (1.628–3.832)	< 0.001	1.820 (1.168–2.834)	0.008
AFP (ng/mL), ≤ 20 vs > 20	2.509 (1.611–3.906)	< 0.001	1.750 (1.105–2.771)	0.017
DCP (mAU/mL), ≤ 40 vs > 40	2.199 (1.414–3.422)	< 0.001	1.936 (1.219–3.075)	0.005
TB (μmol/L), ≤ 20.4 vs > 20.4	1.073 (0.686–1.679)	0.757		
ALT (U/L), ≤ 50 vs > 50	0.645 (0.350–1.190)	0.161		
AST (U/L), ≤ 40 vs > 40	0.648 (0.388–1.083)	0.098	0.692 (0.409–1.171)	0.170
Alb (g/L), ≤ 35 vs > 35	1.616 (0.910–2.870)	0.101		
GGT (U/L), ≤ 60 vs > 60	1.102 (0.713–1.703)	0.662		
ALP (U/L), ≤ 125 vs > 125	0.769 (0.424–1.394)	0.387		
CR (μmol/L), ≤ 104 vs > 104	1.688 (0.531–5.367)	0.375		
INR, ≤ 1.20 vs > 1.20	1.623 (0.811–3.249)	0.172		
Maximum tumor diameter (mm), ≤ 20 vs > 20	2.386 (1.525–3.733)	< 0.001	1.813 (1.119–2.939)	0.016
Number of tumors, single vs multiple	2.726 (1.727–4.302)	< 0.001	1.766 (1.082–2.883)	0.023

HCC, Hepatocellular carcinoma; RFA, Radiofrequency ablation; CTC, Circulating tumor cell; AFP, Alpha-fetoprotein; DCP, Des-γ-carboxy prothrombin; TB, Total bilirubin; ALT, Alanine aminotransferase; AST, Aspartate aminotransferase; Alb, Albumin; GGT, Gamma-glutamyltransferase; ALP, Alkaline phosphatase; CR, Creatinine; INR, International normalized ratio; HR, Hazard ratio; Cl, Confidence interval.

### Recurrence curve of independent risk factors for tumor recurrence

**Figure 2:**
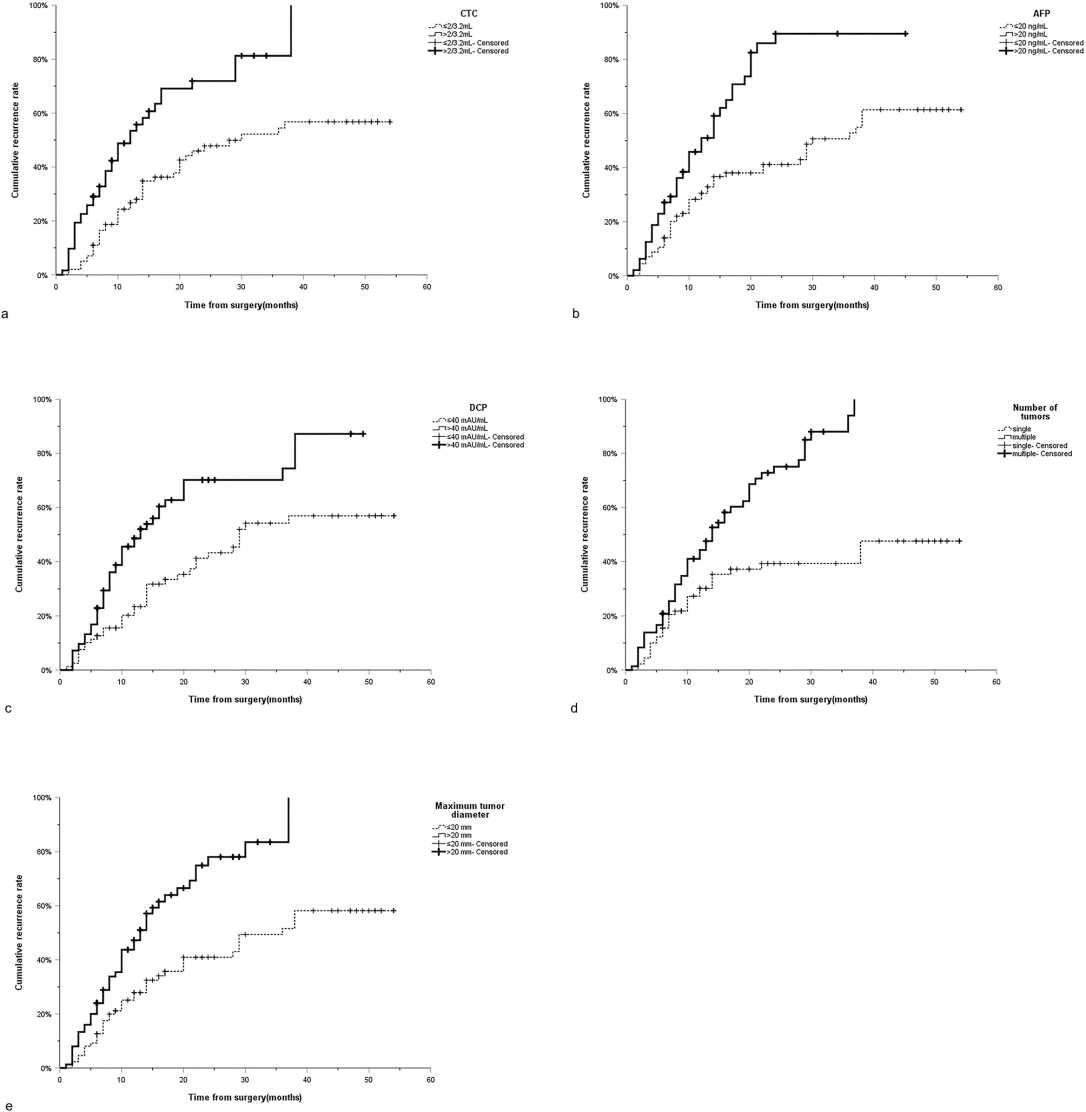
Curves of cumulative recurrence rate of RFA of HCC of (**a**) CTC, (**b**) AFP, (**c**) DCP, (**d**) number of tumors and (**e**) maximum tumor diameter in overall cohort. RFA Radiofrequency ablation; HCC Hepatocellular carcinoma; CTC Circulating tumor cells; AFP Alpha-fetoprotein; DCP Des-γ-carboxy prothrombin.

**Figure 3:**
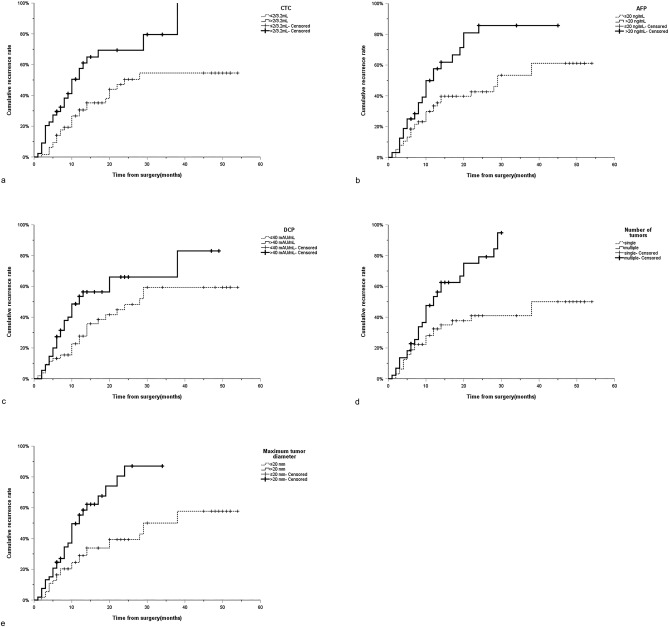
Curves of cumulative recurrence rate of RFA of HCC of (**a**) CTC, (**b**) AFP, (**c**) DCP, (**d**) number of tumors and (**e**) maximum tumor diameter in derivation cohort. RFA Radiofrequency ablation; HCC Hepatocellular carcinoma; CTC Circulating tumor cells; AFP Alpha-fetoprotein; DCP Des-γ-carboxy prothrombin.

**Figure 4:**
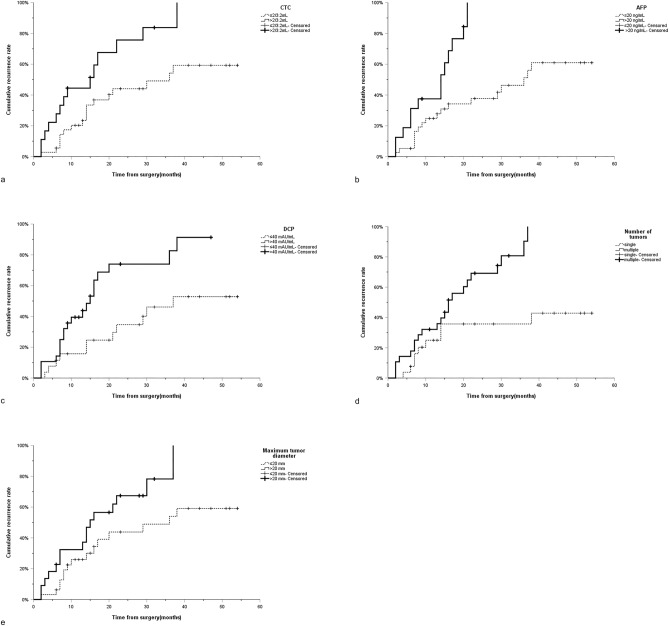
Curves of cumulative recurrence rate of RFA of HCC of (**a**) CTC, (**b**) AFP, (**c**) DCP, (**d**) number of tumors and (**e**) maximum tumor diameter in validation cohort. RFA Radiofrequency ablation; HCC Hepatocellular carcinoma; CTC Circulating tumor cells; AFP Alpha-fetoprotein; DCP Des-γ-carboxy prothrombin.

The median follow-up time for patients with HCC after percutaneous RFA was 12 months (range 1–54 months). During the follow-up period, 86 patients had tumor recurrence, 76 patients had no recurrence, and the 1-, 2- and 3-year cumulative recurrence rates were 36.6%, 56.5% and 64.7%, respectively. In the Kaplan–Meier analysis of the overall cohort cohort, the median time of tumor recurrence in patients with low CTC (≤ 2/3.2 mL) was 30 months (95%CI:14.6–45.4 months), while that in patients with high CTC (> 2/3.2 mL) was 12 months (95%CI:8.0–16.0 months) (*P* < 0.001) (Fig. 2a); The median time of tumor recurrence in patients with low AFP (< 20 ng/mL) was 30 months (95%CI:23.8–36.2 months), while that in patients with high AFP (> 20 ng/mL) was 12 months (95%CI:8.4–15.6 months) (*P* < 0.001) (Fig. 2b); The median time of tumor recurrence in patients with low DCP (≤ 40 mAU/mL) was 29 months (95%CI:16.4–41.6 months), while that in patients with high DCP (> 40 mAU/mL) was 13 months (95%CI:9.0–17.0 months) (*P* < 0.001) (Fig. 2c); Patients with single tumor number do not have a median recurrence time, while patients with multiple tumor numbers have a median tumor recurrence time of 14 months (95%CI:10.5–17.5 months) (*P* < 0.001) (Fig. 2d); The median time of tumor recurrence in patients with maximum tumor diameter ≤ 20 mm was 36 months (95%CI:27.7–44.4 months), while in patients with maximum tumor diameter > 20 mm, the median time of tumor recurrence was 13 months (95%CI:9.3–16.7 months) (*P* < 0.001) (Fig. 2e). In addition, Kaplan–Meier analysis in the derivation cohort also showed that CTC > 2/3.2 mL (*P* = 0.001), AFP > 20 ng/mL (*P* = 0.004), DCP > 40 mAU/mL (*P* = 0.016), the number of multiple tumors (*P* < 0.001) and maximum tumor diameter > 20 mm (*P* = 0.001) are independent risk factors for tumor recurrence (Fig. 3a–e). Similarly, similar results were found in the validation cohort (CTC:*P* = 0.007; AFP:*P* = 0.001; DCP:*P* = 0.005; Number of tumors:*P* = 0.003; Maximum tumor diameter:*P* = 0.037) (Fig. 4a–e). It can be seen that CTC, AFP, DCP, maximum tumor diameter and tumor number are powerful predictors of tumor recurrence after percutaneous radiofrequency ablation of HCC.

### Recurrence curve of scoring system and risk group

**Figure 5:**
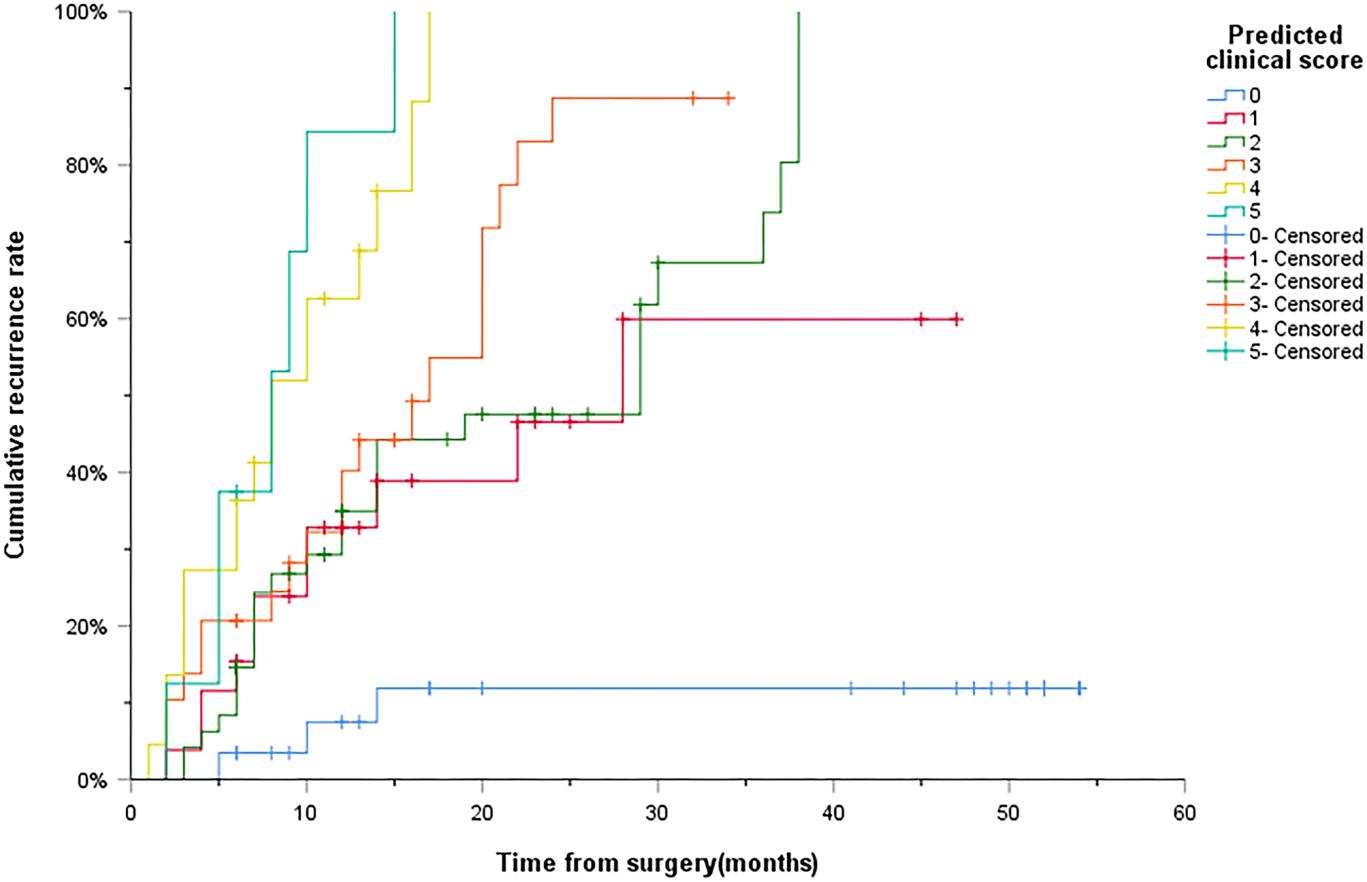
Preoperative clinical score 0 to 5 scores to predict the cumulative recurrence rate curve of HCC after RFA. RFA Radiofrequency ablation; HCC Hepatocellular carcinoma.

**Figure 6:**
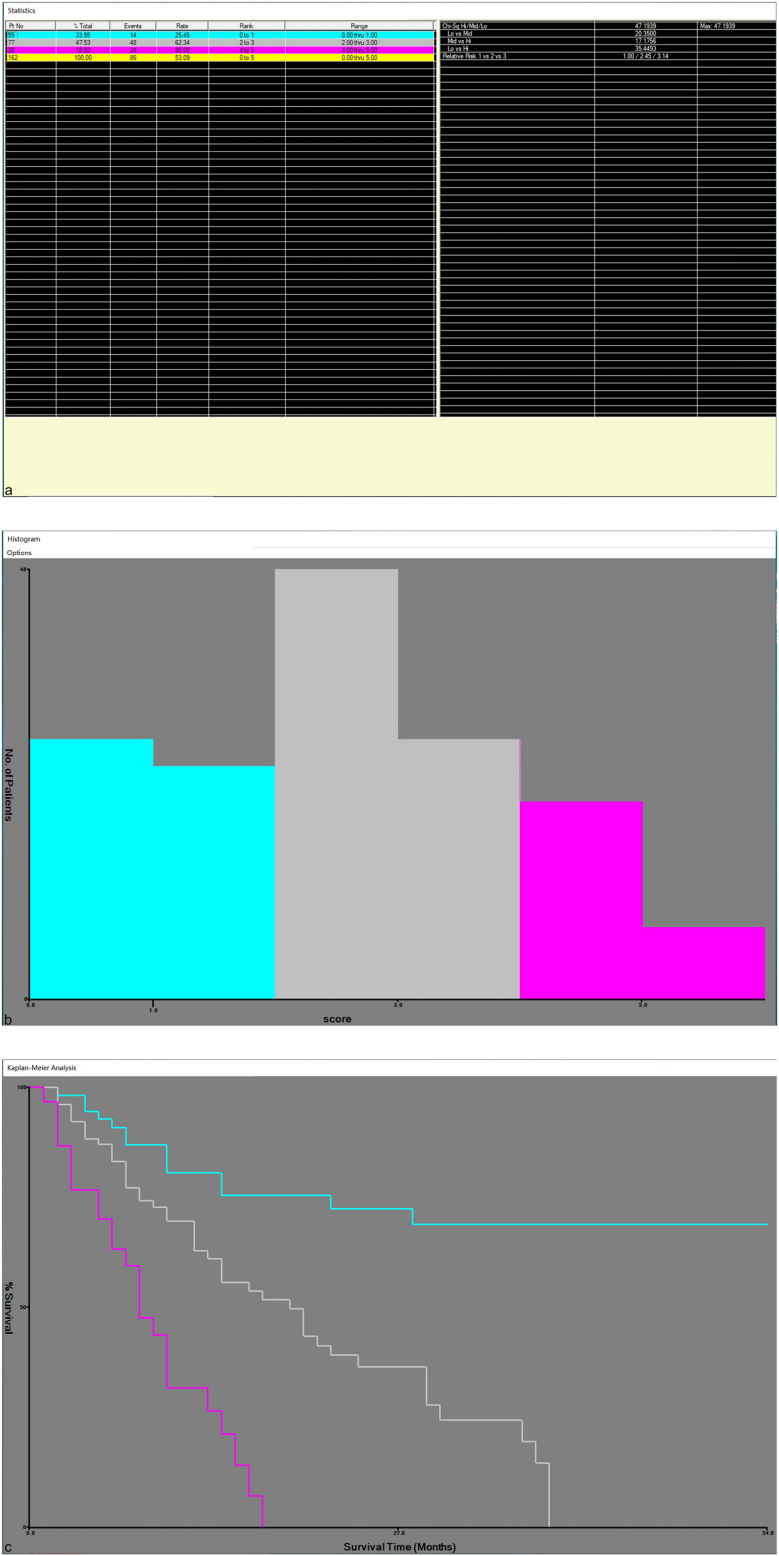
X-tile plots of predicted clinical scoring system and tumor-free survival of patients with HCC after percutaneous RFA. X-tile plots showing χ^2^ values with cut-off points to generate Low-risk, Medium-risk and High-risk groups. (**a**) The optimal cut-off value of the clinical scoring system was 1 score and 3 scores at the maximum χ^2^ value of 47.1939. (**b**) Histogram of the entire cohort divided into Low-risk, Medium-risk and High-risk groups according to the optimal cut-off value of 1 score and 3 scores. Blue bars represent the Low-risk group, grey bars represent the Medium-risk group, and red bars represent the High-risk group. (**c**) Kaplan–Meier plot of tumor-free survival in groups stratified using the optimal cut-off value of predicted clinical scoring system. Blue curves represent the Low-risk group, grey curves represent the Medium-risk group, and red curves represent the High-risk group. RFA Radiofrequency ablation; HCC Hepatocellular carcinoma.

**Figure 7:**
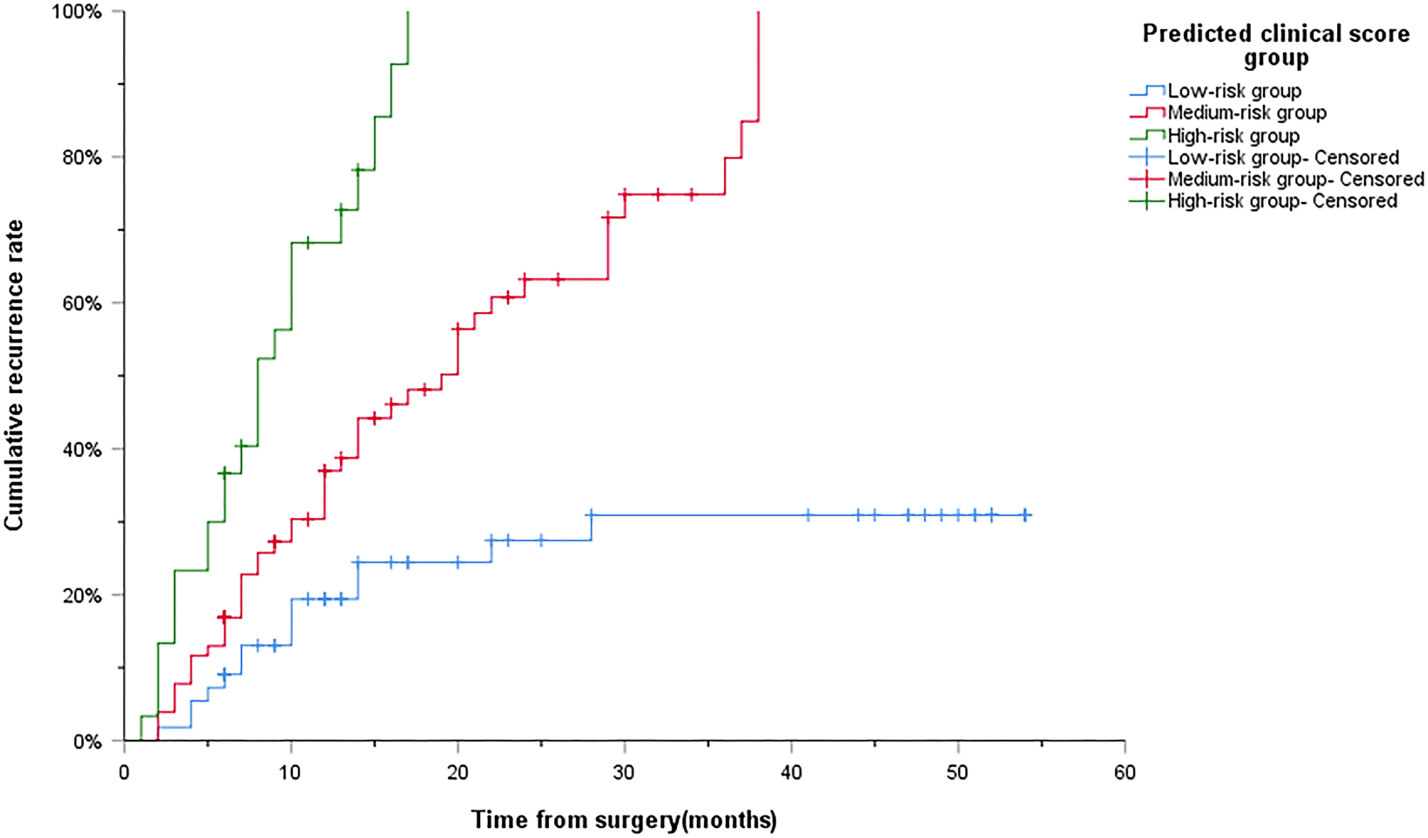
Preoperative clinical score grouping to predict the cumulative recurrence rate curve after RFA of HCC. Low-risk group: 0 to 1 score; Medium-risk group: 2 to 3 scores; High-risk group: 4 to 5 scores. RFA Radiofrequency ablation; HCC Hepatocellular carcinoma.

Considering that the hazard ratio of the five independent risk factors affecting tumor recurrence are similar, we assign a score of 1 to each independent risk factor, and Kaplan–Meier analysis is used to draw recurrence curves with different scores. As shown in Fig. 5, the median time of tumor recurrence was not shown in patients with 0 scores. The median time of tumor recurrence in patients with 1, 2, 3, 4 and 5 scores was 28 months, 29 months, 17 months, 8 months and 8 months, respectively. On the basis of our designed predictive clinical scoring system with a score of 0 to 5, X-Tiles software was used to divide the score into a low-risk group (0 scores–1 score), a medium-risk group (2 scores–3 scores), and a high-risk group (4 scores–5 scores) (Fig. 6a–c). Among these three groups, the cumulative tumor recurrence rates of patients in low-risk group, medium-risk group and high-risk group in 1 year, 2 years and 3 years were 19.4%/27.5%/30.9%, 37.0%/63.2%/79.9% and 68.2%/100%/100%, respectively. There is a significant difference between them (low-risk group vs medium-risk group: *P* < 0.001; medium-risk group vs high-risk group: *P* < 0.001) (Fig. 7).

## Discussion

In order to optimize the indications before RFA of HCC and reduce tumor recurrence after RFA, many studies have made a significant contribution to the analysis of risk factors for recurrence after RFA^5,10–13^. Lin et al. and Yang et al. have also confirmed that HCC patients with tumor diameter > 20 mm and multiple tumor nodules have a significantly increased risk of tumor recurrence after RFA^18,19^. AFP and DCP are a wide range of tumor markers closely related to HCC, which are often used in the diagnosis of HCC and the prognosis of tumor recurrence^5,13^. It can be seen that more and more evidence shows that there is a strong correlation between tumor biomarkers and HCC, and the interaction between tumor itself and tumor biomarkers can lead to tumor development^10,11,13,20,21^.

What is noteworthy is that we found that CTC is one of the important independent risk factors for tumor recurrence after RFA in this research. CTC is a tumor cell that metastases from the primary tumor to the blood or lymphatic system, and then locates in the blood, bone marrow, lymph nodes and other healthy organs. Its existence is the process of tumor growth and distant formation of metastatic foci^22,23^. It can be seen that CTC has similar or the same biological characteristics as the primary tumor, and the "liquid biopsy" of the primary tumor and metastatic focus can be realized by testing the blood, which causes its role in malignant tumors attracting more and more attention^24,25^. At present, as a marker reflecting tumor invasion, CTC has long been used in the evaluation of curative effect, individual treatment and prognosis monitoring of malignant tumors such as breast cancer, lung cancer and colorectal tumor^16,25,26^. Sun et al. and Wang et al. have revealed that the higher the level of peripheral CTC in patients with HCC, the higher the risk of tumor recurrence after hepatectomy, which reflects that CTC can be used as a predictor of postoperative recurrence of HCC^27,28^. In addition, we analyzed that CTC was positively correlated with maximum tumor diameter, the number of tumors and AFP levels in the independent prognostic risk factors in this study, and scholars have previously confirmed the accuracy of this conclusion, which further demonstrated the prognostic value of CTC in patients with HCC after RFA^29^.

Some scholars have previously reported that the more the number of high-level tumor markers, the greater the risk of microvascular invasion in patients with HCC, which will lead to a significantly higher tumor recurrence rate after RFA compared with hepatectomy^30^. In addition, Nitta et al. and Ueno et al. found that RFA itself became a prognostic risk factor for HCC when two or three tumor markers were highly expressed^5,31^. Therefore, RFA alone may not be suitable for high-risk group with high levels of expression of at least two or three tumor markers in patients with 3 cm or smaller HCC. In this study, we learned that the hazard ratio of risk factors for recurrence after RFA are similar, so we designed this simple clinical scoring system. Compared with the prediction models of other studies, our clinical scoring system not only discards complicated calculation formulas, but also has a certain degree of scientificity in predicting the recurrence after RFA before treatment^10,32^. Therefore, in clinical practice, RFA should be carefully selected to treat patients with HCC in the middle and high risk group.

## Study limitation

In this study, as a retrospective analysis with a small sample size, our clinical scoring system is not only lack of external review, but also unable to analyze the specific combination of three tumor markers to assess the risk of tumor recurrence. This makes our study have great limitations. It is hoped that there will be a large number of clinical studies to verify our clinical scoring risk model in the future, so that more patients can get timely, reasonable and effective treatment.

## Conclusion

To sum up, in patients with 3 cm or less HCC, based on CTC > 2/3.2 mL, AFP > 20 ng/mL, DCP > 40mAU/mL, maximum tumor diameter > 20 mm and multiple tumor numbers (≥ 2), we designed a simple clinical scoring system to predict tumor recurrence after RFA, which has a certain application value for preoperative clinical decision-making.
